# Targeting the DNA Damage Response to Increase Anthracycline-Based Chemotherapy Cytotoxicity in T-Cell Lymphoma

**DOI:** 10.3390/ijms23073834

**Published:** 2022-03-30

**Authors:** Martina Magni, Chiara Paolizzi, Chiara Monfrini, Cristina Vella, Paolo Corradini, Cristiana Carniti

**Affiliations:** 1 Department of Medical Oncology and Hematology, Fondazione IRCCS Istituto Nazionale dei Tumori, Via Venezian 1, 20133 Milan, Italy; martina.magni@istitutotumori.mi.it (M.M.); chiarapaolizzi@gmail.com (C.P.); chiara.monfrini@istitutotumori.mi.it (C.M.); cristina.vella@istitutotumori.mi.it (C.V.); paolo.corradini@unimi.it (P.C.); 2 School of Medicine, Università degli Studi di Milano, Via Venezian 1, 20133 Milan, Italy

**Keywords:** T-cell lymphoma, anthracycline-based chemotherapy, DNA damage response, ATM inhibition, AZD0156, drug combination

## Abstract

Mature T-cell lymphomas (MTCLs) represent a heterogeneous group of aggressive non-Hodgkin lymphomas comprising different entities. Anthracycline-based regimens are considered the standard of care in the front-line treatment. However, responses to these approaches have been neither adequate nor durable, and new treatment strategies are urgently needed to improve survival. Genomic instability is a common feature of cancer cells and can be caused by aberrations in the DNA damage response (DDR) and DNA repair mechanisms. Consistently, molecules involved in DDR are being targeted to successfully sensitize cancer cells to chemotherapy. Recent studies showed that some hematological malignancies display constitutive DNA damage and intrinsic DDR activation, but these features have not been investigated yet in MTCLs. In this study, we employed a panel of malignant T cell lines, and we report for the first time the characterization of intrinsic DNA damage and basal DDR activation in preclinical models in T-cell lymphoma. Moreover, we report the efficacy of targeting the apical kinase ATM using the inhibitor AZD0156, in combination with standard chemotherapy to promote apoptotic cell death. These findings suggest that DDR is an attractive pathway to be pharmacologically targeted when developing novel therapies and improving MTCL patients’ outcomes.

## 1. Introduction

Nodal T-cell lymphomas (TCL) are a rare and aggressive subgroup of lymphoid malignancies, accounting for 10–15% of non-Hodgkin lymphomas (NHL). According to the latest World Health Organization (WHO) classification, they comprise several entities, such as angioimmunoblastic T-cell lymphoma (AITL), anaplastic large T-cell lymphoma (ALCL), and peripheral T-cell lymphoma not otherwise specified (PTCL-NOS), which is the most common subtype including all the TCL cases that still fail to be categorized [1,2]. Anthracycline-based chemotherapies—namely, CHOP-based (cyclophosphamide, doxorubicin, vincristine and prednisone) or CHOEP-based (CHOP + etoposide) regimens—are commonly used as front-line approaches. Suboptimal results led investigators to design preclinical and clinical studies to test the addition of novel agents, monoclonal antibodies or kinase inhibitors to these regimens to improve efficacy [3,4]. In this view, we recently reported the benefits of adding the tyrosine kinase inhibitor dasatinib to CHOEP [5]. Nonetheless, with the exception of ALK+ ALCL, in real-life experience, responses and overall survival (OS) rates for patients with TCL are still very low [4]. Thus, there is need to increase our knowledge of TCLs biology to design innovative treatment strategies and improve outcomes.

In recent years, several efforts have been made by us and others to elucidate the genetic features of TCLs, reporting a broad range of copy number alterations (CNAs) and structural variations (SVs) rather than point mutations [6,7,8], thus suggesting that TCLs are characterized by genomic instability. It is well consolidated that genomic instability is a hallmark of cancer cells [9,10,11] that has been recently described also in hematological malignancies including multiple myeloma (MM), diffuse large B cell lymphoma (DLBCL), acute myeloid leukemia (AML), and chronic lymphocytic leukemia (CLL) [12,13,14,15,16,17,18] but has never been investigated in TCLs. A peculiarity of genomic instability is the presence of intrinsic DNA damage usually associated with basal activation of the DNA damage response (DDR). One of the key players of DDR is the serine/threonine kinase ataxia-telangiectasia mutated (ATM), an apical kinase and a sensor of DNA damage. In the presence of DNA lesions, ATM is one of the first molecules to be activated in order to spread the signal throughout the nucleus. The signaling cascade triggered by ATM mediates cell cycle checkpoints activation and promotes the recruitment of specialized proteins to sites of damage to allow DNA repair [19]. Because of its role as master regulator of DDR signaling, ATM is an appealing therapeutic target for successfully sensitizing cancer cells to standard treatments [20]. Accordingly, inhibitors of ATM and other DDR signaling kinases are now being tested in clinical trials either as monotherapy for specifically mutated tumors or in combination with chemotherapeutic agents [21,22]. AZD0156, a recently developed selective ATM inhibitor [23], was able to enhance the effects of olaparib and radiation in preclinical models of different solid tumors [24,25] and is now being tested in advanced solid tumors alone in or in combination with olaparib and irinotecan-based chemotherapy in a phase I study (NCT02588105).

Here, we report for the first time the characterization of intrinsic DNA damage and basal DDR activation in preclinical models in T-cell lymphoma. Additionally, we provide evidence that targeting the apical kinase ATM in combination with CHOEP is effective in promoting apoptotic cell death.

## 2. Results

### 2.1. Malignant T Cell Lines Are Characterized by Endogenous Levels of DNA Damage and Basal Activation of DDR Signaling

**Figure 1:**
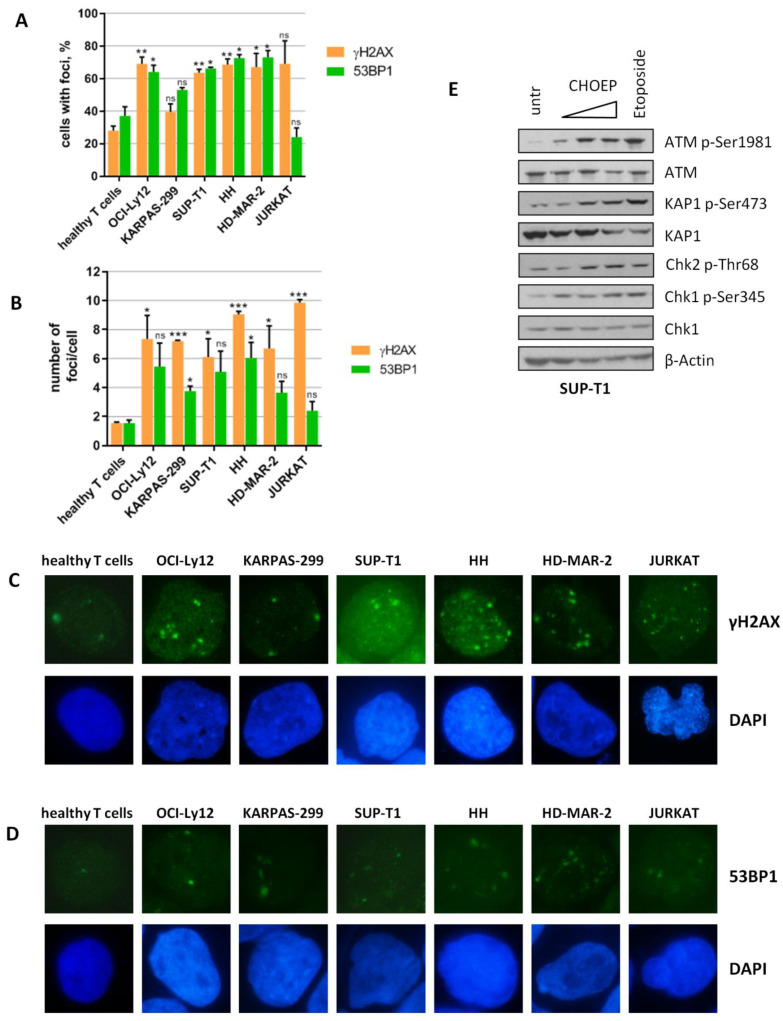
TCL cell lines show intrinsic DNA damage and basal activation of DDR. Healthy T lymphocytes isolated from healthy donors and malignant T cell lines (OCI-Ly12, KARPAS-299, SUP-T1, HH, HD-MAR-2, and JURKAT) were subjected to immunofluorescence for the evaluation of γH2AX or 53BP1 foci (**A**,**B**). The percentage (%) of cells displaying foci and the number of foci/cell in the subsets of cells with detectable foci are reported in (**A**,**B**), respectively. Data are expressed as the mean ± SD of independent experiments. Asterisks indicate statistically significant differences between each cell line and healthy T lymphocytes (* *p* < 0.05; ** *p* < 0.01; *** *p* < 0.001; ns: not significant). Representative immunofluorescence images (**C**,**D**) of experiments described in (**A**,**B**), original microscope magnification 100×. SUP-T1 cells were exposed to increasing concentrations of CHOEP (IC_50_, 4× and 8×, as in [5]) or to 20 μM etoposide for 3 h. After harvesting, untreated and treated cell lysates were analyzed by Western blot using the indicated antibodies (**E**).

We first explored whether T-cell lymphomas as well as solid tumors and some hematological malignancies are characterized by intrinsic DNA damage, a feature of genomic instability. The presence of γH2AX and 53BP1 foci, well-recognized markers of DNA damage [26,27], was evaluated by immunofluorescence in a panel of cancer cell lines of T lineage representing the heterogeneity of TCLs. We observed that all malignant T cells evaluated, with the exception of KARPAS-299 and JURKAT, display a higher percentage of nuclei with detectable γH2AX and 53BP1 foci compared with normal T lymphocytes isolated from the peripheral blood of healthy donors (62.8 ± 12.3 vs. 28 ± 2.8 for γH2AX foci; 58.8 ± 17.9 vs. 37 ± 5.7 for 53BP1 foci; mean ± SD of all malignant T cells vs. healthy T cells, respectively) (Figure 1A). In addition, the number of γH2AX foci/cell was significantly higher in all cell lines than in normal T lymphocytes (7.7 ± 1.6 vs. 1.55 ± 0.07; mean ± SD of all malignant T cells vs. healthy T cells, respectively) (Figure 1B,C). The number of 53BP1 foci/cell, although higher than in normal T lymphocytes in all TCL cell lines, was significantly superior only in two out of six cell lines (KARPAS-299 and HH) (Figure 1B,D). As expected, 24 h upon chronic treatment with the anthracycline-based chemotherapy regimen CHOEP, the presence of both γH2AX and 53BP1 foci significantly increased (Figure S1), consistent with the fact that CHOEP components are well-established DNA damaging agents [28,29,30,31].

We then assessed whether the presence of intrinsic DNA damage was associated with constitutive activation of DDR signaling pathways. To this aim, we studied the expression of activating post-translational modifications of several DDR players including ATM, Chk2, Chk1, and KAP1 before and after 3 h treatment with increasing concentrations of CHOEP and with 20 µM etoposide alone, which is well known to activate DDR in human cells [30,32]. As shown in Figure 1E and Figure S2, TCL cell lines display basal DDR activation, which is further enhanced upon chemotherapy exposure. Basal DDR activation was not present (data not shown) as expected in healthy T lymphocytes [33,34].

Taken together, these data indicate that in vitro models of T-cell lymphoma show intrinsic DNA damage and constitutive activation of the DDR signaling pathways, despite some degree of heterogeneity observed in the analyzed cell lines.

### 2.2. Preclinical Models of T-Cell Lymphoma Are Sensitive to the ATM Inhibitor AZD0156

As we have shown that the DDR signaling cascade is basally activated and gets further triggered in response to chemotherapy, we hypothesized that targeting the DDR could block the possible attempts made by the cell exposed to CHOEP to repair the induced DNA lesions, thus leading to augmented apoptosis. For this purpose, we took advantage of a recently developed inhibitor, AZD0156, that targets the apical kinase ATM [23] and is being tested in clinical trials for solid tumors.

**Figure 2:**
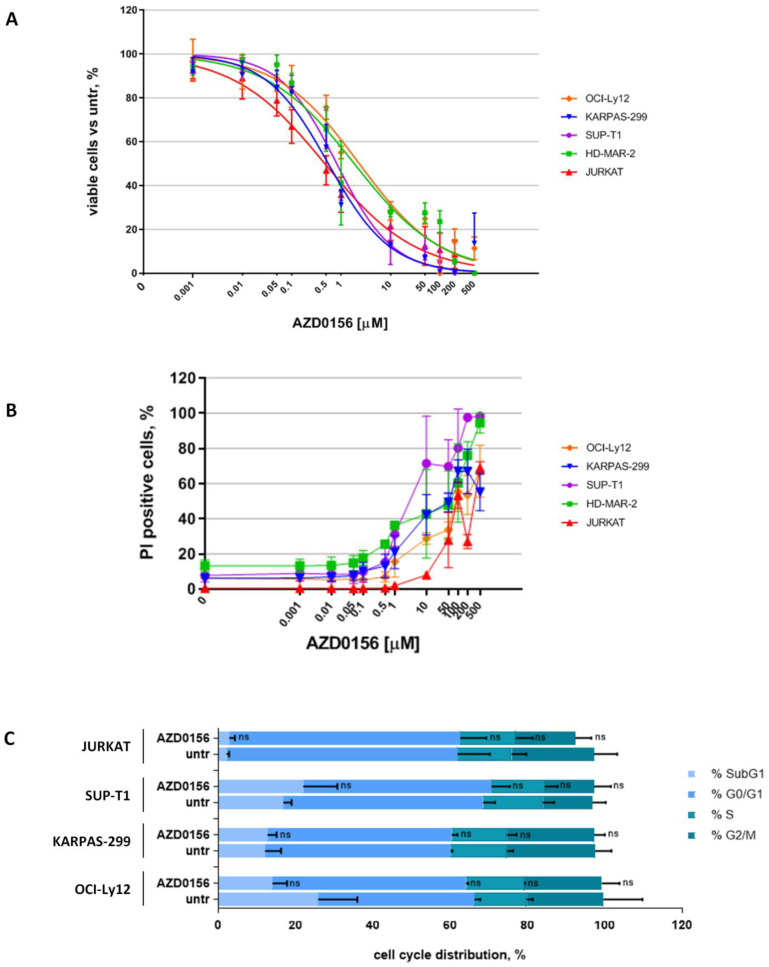
ATM inhibitor AZD0156 has a cytotoxic effect on malignant T cells. Cell lines were exposed to increasing concentrations of AZD0156 (range 1 nM–500 µM) for 48 h. Then cells were studied by flow cytometry. Titration curves are reported (**A**), dead cells (PI-positive) (**B**), and cell cycle distribution (**C**) were monitored. In (**A**,**B**), data are expressed as percentage of untreated samples and are the mean ± SD of at least four independent experiments. In (**C**), data are the mean ± SD of at least two independent experiments (ns: not significant).

First, we exposed cell lines to increasing concentrations of AZD0156 (range 1 nM–500 µM) for 48 h and observed a dose-dependent reduction in viable cells in all cell lines, even though sensitivity to AZD0156 was heterogeneous (Figure 2A). The only exception was represented by HH cells, which displayed only a mild reduction in cell viability when exposed to high doses of AZD0156 (not shown) and that for this reason were excluded from subsequent experiments. We then calculated the inhibitory concentrations (ICs) for each cell line (IC_50_ range: 0.55–2.3 µM) (Table S1). The decrease in cell viability observed was caused by an increase in dead cells (which internalize propidium iodide, PI), thus suggesting a cytotoxic rather than a cytostatic effect of this compound on the tested cell lines (Figure 2B). This was further corroborated by cell cycle analyses upon IC_20_ (Figure 2C and Figure S3) and IC_50_ (not shown) doses of AZD0156 with no significant changes detected among untreated and AZD0156-treated cells.

### 2.3. ATM Inhibition Sensitizes Malignant T Cell Lines to CHOEP Treatment

**Figure 3:**
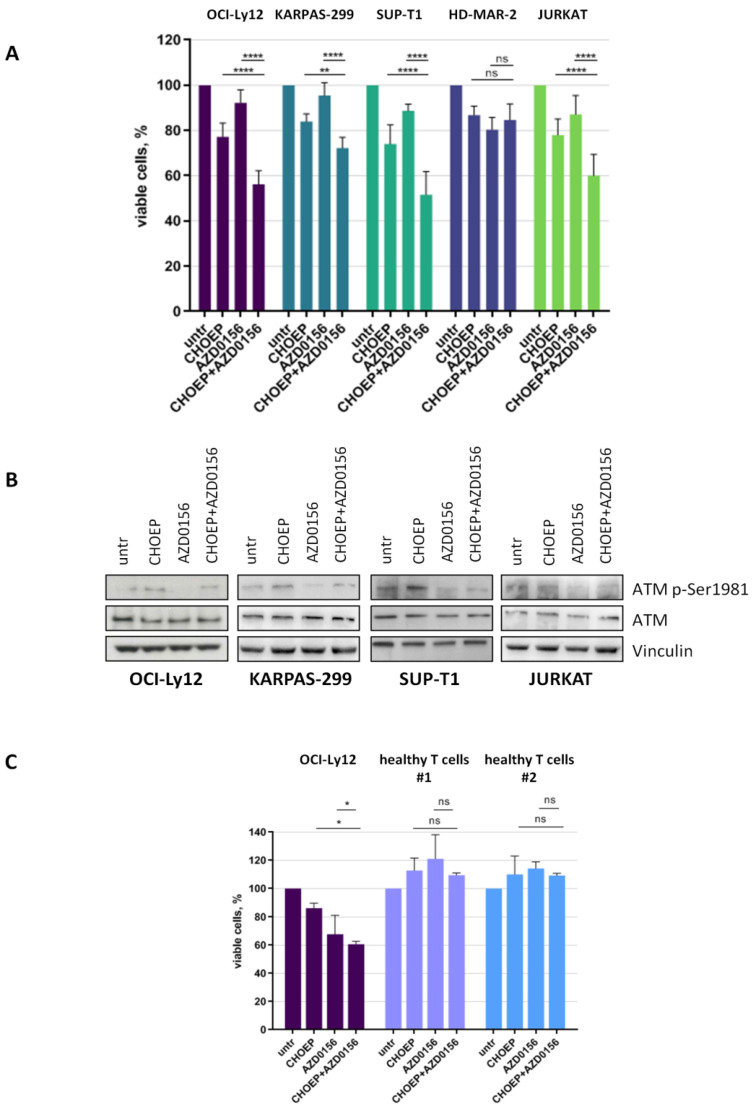
AZD0156 promotes CHOEP-induced reduction in cell viability in malignant T cell lines. The indicated cell lines were treated with IC_20_ CHOEP and IC_20_ AZD0156 alone or in combination for 48 h. Then cell viability was assessed by flow cytometry (**A**), and cell lysates were subjected to Western blot using the indicated antibodies (**B**). OCI-Ly12 and healthy T lymphocytes, isolated from the peripheral blood of two different healthy donors, were treated for 48 h with IC_20_ CHOEP and IC_20_ AZD0156 alone or in combination. Then viable cells were monitored by flow cytometry (**C**). In A and C, data are expressed as the percentage of untreated samples and are the mean ± SD of at least three independent experiments (* *p* < 0.05; ** *p* < 0.01; **** *p* < 0.0001; ns: not significant).

We then decided to exploit malignant T cell sensitivity to AZD0156 to potentiate CHOEP cytotoxic effects. Thus, we exposed malignant T cell lines to IC_20_ CHOEP (defined in our previously reported study [5]) and IC_20_ AZD0156 (Table S1), alone and in combination, and we monitored cell growth by flow cytometry. With the exception of HD-MAR-2 cells, in all cell lines the addition of AZD0156 to CHOEP significantly reduced cell proliferation, compared with CHOEP treatment alone (CHOEP 81.5 ± 6.4; CHOEP + AZD0156 68.1 ± 13.6; mean ± SD of all cell lines, *p* < 0.05) (Figure 3A). Consistent with these findings, CHOEP treatment caused an induction of ATM-Ser1981 auto-phosphorylation, which was abrogated by AZD0156 addiction, indicating the molecular activity of the ATM inhibitor (Figure 3B). Importantly, AZD0156 used either alone or combined, as well as CHOEP [5], did not impair cell viability of normal T lymphocytes (Figure 3C).

To rule out the possibility that the different effects observed in the cell lines treated with either AZD0156 alone or the AZD0156 + CHOEP combination could be due to the presence of mutations in *ATM* and/or *TP53*, we performed targeted sequencing analysis. As expected, all cell lines bear pathogenic *TP53* alterations but no *ATM* mutations. *ATM* is mutated only in JURKAT cell line, but we were unable to define a clear inactivating effect of the detected alterations (Tables S2 and S3).

When monitoring γH2AX foci upon treatment with AZD0156 and CHOEP, alone and in combination, a basal level of DNA damage foci was observed in untreated samples, which markedly increased upon CHOEP treatment (Figure S4A). In AZD0156 treated cells, the number of foci was comparable with that of untreated cells, possibly because γH2AX (H2AX-phospho-Ser139) is phosphorylated by ATR and DNA-PK too [26]. Of note, the combination of CHOEP and the ATM inhibitor was able to prevent CHOEP-induced γH2AX phosphorylation. These data are consistent with the fact that γH2AX is a direct target of ATM, which is affected by ATM inhibition. As expected, when monitoring 53BP1 foci upon CHOEP and AZD0156 treatment, we observed that both CHOEP given alone and the CHOEP–AZD0156 combination cause the induction of 53BP1 foci formation irrespective of ATM inhibition (Figure S4B). We also monitored the signaling downstream ATM in response to treatment with AZD0156 and CHOEP. As expected, the treatment with AZD0156 given either alone or in combination with CHOEP impacts not only ATM activation, as shown in Figure 3B, but also its activity on downstream targets (Figure S5).

### 2.4. ATM Inhibitors AZD0156 and KU-55933 Enhance CHOEP-Induced Apoptosis

**Figure 4:**
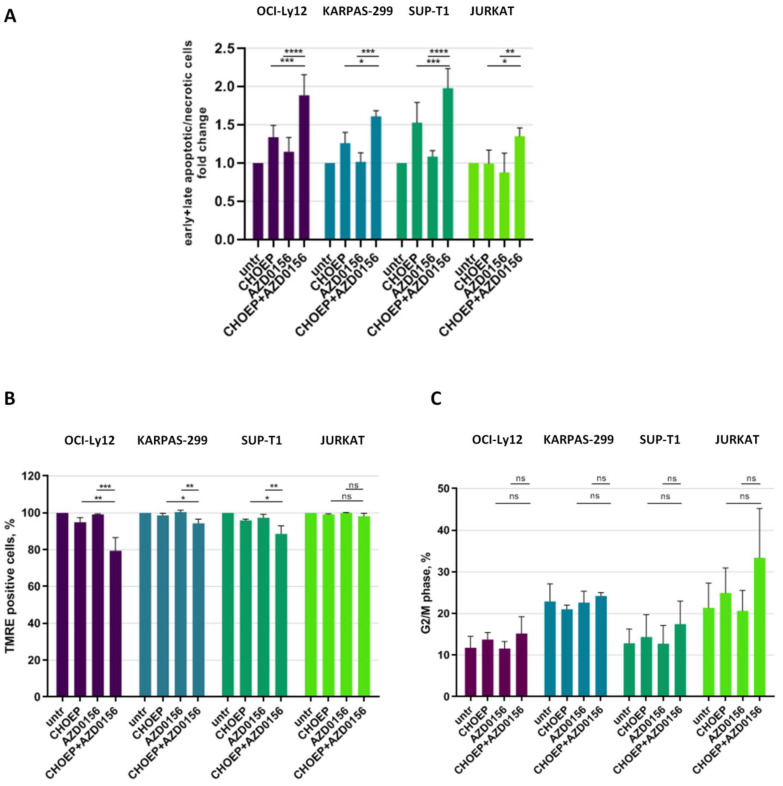
AZD0156 promotes CHOEP-induced apoptosis. The indicated cell lines were treated with IC_20_ CHOEP and IC_20_ AZD0156 alone or in combination for 48 h. Then, cells were stained with the FITC–Annexin V/PI kit to monitor cell death (**A**) or with the fluorescent probe TMRE for the mitochondrial membrane depolarization assessment (**B**) and monitored by flow cytometry. In (**A**), early apoptotic cells are annexin V positive/PI negative, late apoptotic cells are annexin V positive/PI positive and necrotic cells are PI positive. Additionally, cell cycle distribution was assessed by flow cytometry (**C**). In (**B**) data are expressed as percentage of untreated samples. In (**A**–**C**) data are the mean ± SD of at least three independent experiments. Asterisks indicate statistically significant differences (* *p* < 0.05; ** *p* < 0.01; *** *p* < 0.001; **** *p* < 0.0001; ns: not significant).

We further investigated the mechanisms responsible for the reduction in cell proliferation caused by the addition of AZD0156 to CHOEP, monitoring cell death and cell cycle by flow cytometry. We observed that, compared with CHOEP alone, the AZD0156–CHOEP combination caused an increase in apoptotic/necrotic cell death in all cell lines (fold change to untreated cells: CHOEP 1.31 ± 0.28; CHOEP + AZD0156 1.75 ± 0.33; mean ± SD of all cell lines, *p* < 0.0001) (Figure 4A), with a partial involvement of mitochondrial membrane depolarization, an event associated with apoptosis (Figure 4B). As expected, when monitoring cell cycle perturbations, we observed a trend towards increased cell cycle arrest in G2/M phase when we combined AZD0156 with CHOEP, compared with CHOEP alone (percentage (%) of cells in G2/M phase: CHOEP 18.94 ± 6.76; CHOEP + AZD0156 23.64 ± 10.86; mean ± SD in all cell lines, *p*: ns) (Figure 4C and Figure S6). The other cell cycle phases were not subjected to substantial modifications compared with CHOEP (not shown). Although not statistically significant, together with the induction of apoptotic cell death, this increase in G2/M arrested cells well explains the reduction in cell growth we observed (Figure 3A).

To conclude, we confirmed the efficacy of ATM inhibition in potentiating CHOEP effects using another ATM inhibitor, KU-55933, widely used in in vitro studies.

We tried to titrate KU-55933 in our cell lines, but in a range of concentrations between 16 nM and 16 μM, we observed a reduction in cell viability of 10–20%. Cell viability was significantly reduced when we reached higher concentration (100 μM–200 μM) (data not shown). As KU-55933 has been administered to cell lines at 10 μM to inhibit ATM kinase activity in other studies [35,36], we decided to adopt this concentration for the reported experiments.

We first exposed cell lines to 10 μM KU-55933 alone and in combination with IC_20_ CHOEP, and we observed a significant reduction in cell proliferation (Figure S7A). Apoptosis induction was confirmed by cleaved caspase 3 expression by Western blot (Figure S7B) and further confirmed by mitochondrial membrane depolarization assay (Figure S7C). These data are in line with a previous study showing, in JURKAT cells, the pro-apoptotic activity of KU-55933 when added to etoposide [33]. As well as AZD0156, also KU-55933, used alone or in combination with CHOEP, did not alter the viability of healthy T lymphocytes (Figure S7D).

## 3. Discussion

To the best of our knowledge, this is the first study evaluating the presence of intrinsic DNA damage and basal DDR activation in preclinical models of TCL. This is not surprising, as similar evidence was already reported in solid tumors and in some hematological malignancies—namely, DLBCL, MM, AML, and CLL [12,13,14,15,16,17,18]. Of interest, in MM, Cottini and colleagues reported that DNA damage is caused by replicative stress and identified a subset of patients characterized by chromosomal instability and poor prognosis and correlated these features with an increased expression of the oncogene MYC [12,13]. Interestingly, higher MYC levels have been reported in MTCL patients characterized by a worse clinical course and poor response to therapy [8,37]. Thus, further investigations will be needed to clarify whether MYC overexpression is involved in the establishment of intrinsic DNA damage also in PTCLs.

Recently, we and others have reported high frequencies of *CDKN2A* and *TP53* alterations in PTCLs [6,7,8]. *CDKN2A* encodes for p16^INK4a^, a protein that acts as a tumor suppressor gene and is involved in the modulation of cell cycle progression, thus explaining why *CDKN2A* deletion is a frequent event in cancer establishment [38]. Moreover, we have shown that the genome of MTCL patients is characterized by high genomic instability associated with chromothripsis [6], thus further supporting the role of DNA replication stress in the pathogenesis of MTCLs.

The association between genomic instability and intrinsic DNA damage observed in solid and hematological malignancies raises the question of whether targeting the DDR signaling pathway may be exploited to switch DNA repair and pro-survival mechanisms off in cancer cells exposed to genotoxic agents used as standard chemotherapy. In particular, such strategy is appealing in B-cell lymphomas, as during maturation and antibodies production, B cells experience somatic hypermutation and V(D)J recombination, which expose cells to high levels of DNA damage. For this, in recent years, several inhibitors of DDR proteins—including ATR, DNA-PK, PARP, Chk1, and WEE1, administered alone and in combination with other agents—have been preclinically tested [39]. Consistently, ATR inhibitors have shown strong cytotoxic and in vivo antitumor activity in mantle cell lymphomas (MCL) and DLBCL, regardless of their *TP53*, *MYC*, and *ATM* mutation status [40].

In the present study, we report encouraging preclinical data describing the benefits of ATM inhibition in promoting CHOEP-induced cell death in preclinical models of T-cell lymphoma using two different chemical compounds—AZD0156 and KU-55933. Despite the presence of basal DDR activation and intrinsic DNA damage observed in all the cell lines included in this study, their response to AZD0156 and AZD0156–CHOEP treatment was heterogeneous, further confirming the heterogeneity of the pathology.

Interestingly, we observed a reduction in γH2AX foci upon combined treatment with AZD0156 and CHOEP because this serine is directly phosphorylated by ATM. Nonetheless, 53BP1 foci formation was not impaired by AZD0156 addition to CHOEP in our models. Thus, the decrease in γH2AX foci is not suggestive of the absence of DNA damage, but rather of dysfunctional DDR signaling caused by ATM inhibition. Consistently, DNA damage is not efficiently repaired, leading to apoptotic cell death, as in Figure 4A,B.

The AZD0156 inhibitor is currently clinically tested in advanced solid tumors alone or in combination with olaparib and irinotecan. Moreover, recent preclinical studies reported AZD0156 capability to enhance the genotoxic effects of olaparib and radiation in preclinical models of different solid tumors [24,25]. In accordance with these data, AZD0156 enhances apoptosis induced by CHOEP treatment.

Given alone, AZD0156 does not induce cell cycle perturbations in malignant T cells, as observed in solid tumors [24,25]. Nonetheless, we reported a mild but not significant modulation of G2/M arrest when we combined the ATM inhibitor with CHOEP that could be explained by ATM involvement in S-phase checkpoint. This finding is in agreement with what observed combining AZD0156 with radiation, as a clear cell cycle arrest was not observed [25]. On the contrary the combination with olaparib strongly caused G2/M cell cycle arrest [24], possibly because the effects of CHOEP are more similar to those experienced by cells exposed to radiation than to olaparib.

In recent years, first-line regimens built on a CHOP-like backbone have been studied, but none, including the one combining the histone deacetylase (HDAC) inhibitor romidepsin with CHOP (Ro-CHOP), significantly improved the survival of patients affected by PTCL [41], thus supporting the concept that identifying better treatments remains a major unmet need. In this view, the data we present, indicate that ATM inhibition combined with anthracycline-based programs represents a potential new therapeutic option for the treatment of TCLs.

The in vitro models of T-cell lymphoma employed in this study are characterized by pathogenic *TP53* mutations, suggesting that AZ0156 is effective independent of *TP53* mutational status. On the contrary, we did not observe *ATM* putative inactivating mutations. Nonetheless, consistent with the fact that mutations have been reported in the *ATM* gene in TCL patients [7,42], additional studies will be required to define whether the proposed drug combination can be active in delineated MTCL subtypes and/or if it could be used even in presence of genetic alterations affecting *ATM*.

Notably, our in vitro data suggest that suboptimal concentrations of the ATM inhibitor are not detrimental for healthy T lymphocytes. Nonetheless, we cannot rule out the possibility that the combined treatment with AZD0156 and CHOEP could cause adverse effects in humans. Preliminary data from the phase I clinical trial aimed at assessing efficacy and tolerability of AZD0156 in combination with olaparib reported minor toxicities in about 40% of patients enrolled in the study. However, hematologic toxicities were observed when higher doses of both drugs were used [43]. Thus, phase I studies will be required to explore the safety of the AZD0156–CHOEP combination and to assess the doses and durations of exposure.

## 4. Materials and Methods

### 4.1. Cell Lines

KARPAS-299 (ALCL), SUP-T1 (lymphoblastic TCL), HH (cutaneous TCL), HD-MAR-2 (T-cell leukemia), and JURKAT (T-cell leukemia) were purchased from DSMZ–Leibniz Institute (Braunschweig, Germany). OCI-Ly12 cell line (PTCL-NOS) was a kind gift from Dr. Leandro Cerchietti (Weill Cornell Medicine, New York, NY, USA, as approved by the Ontario Cancer Institute) [44]. Cells were grown as described in [5]. Healthy T lymphocytes were obtained from the peripheral blood of healthy volunteer donors who provided informed consent and were isolated upon density gradient centrifugation and separation with the autoMACS Pro separator (Miltenyi Biotec, Bergisch Gladbach, Germany) using CD3 Micro Beads (Miltenyi Biotec, Bergisch Gladbach, Germany) following the manufacturer’s instructions.

### 4.2. Treatments

All drugs (cyclophosphamide monohydrate, doxorubicin hydrochloride, vincristine sulphate, etoposide, prednisone, and ATM inhibitors AZD0156 and KU-55933) were purchased from Selleck Chemicals (Houston, TX, USA). CHOEP was prepared as described in [5]. Briefly, CHOEP 1× was composed of cyclophosphamide monohydrate 5.84 pM (C), doxorubicin hydrochloride 1.5 pM (H), vincristine sulphate 260 pM (O), etoposide 0.3 μM (E), and prednisone 1 μM (P). AZD0156 was added to cells 30 min before CHOEP. KU-55933 was used at 10 μM and was added to cells 1 h before CHOEP treatment. The half maximal inhibitory concentrations (IC_50_) were determined upon 48 h of chronic exposure, as the concentration of drug able to reduce 50% of cell growth.

### 4.3. Immunofluorescence of γH2AX and 53BP1 Foci

Upon treatment, cells were transferred on glass slides using a cytospin centrifuge (5 min at 500 rpm). Glass slides were dried overnight at room temperature, then fixed with 2% paraformaldehyde and permeabilized with PBS 0.2% Triton X-100. Saturation was performed in PBS 5% BSA 0.2% TWEEN 20. Primary antibodies used were γH2AX #A300-081A (Bethyl Laboratories, Montgomery, TX, USA) and 53BP1 #NB100-305 (Novus Biologicals Bio-Techne, Centennial, CO, USA); secondary conjugated antibody used was Alexa Fluor 488 #A11034 (Thermo Fisher Scientific, Waltham, MA, USA). At the end, nuclei were counterstained with DAPI (Merck Millipore, Burlington, MA, USA). Images were acquired with a Nikon Eclipse E1000 fluorescence microscope equipped with a DSU3 CCD camera, using a 100× magnification objective, as previously described [45]. In each experiment, foci enumeration was performed on at least 100 nuclei.

### 4.4. Cell Viability, Cell Cycle, Cell Death, and Measurement of the Mitochondrial Transmembrane Potential

Cell viability, cell death, mitochondrial membrane potential, and cell cycle were studied by flow cytometry as previously described [5]. Briefly, reagents used to label cells were: propidium iodide (PI, Merck Millipore, Burlington, MA, USA) for cell viability, Annexin V-FITC Kit (Miltenyi Biotec, Bergisch Gladbach, Germany) for cell death, the fluorescent probe tetramethylrhodamine ethyl ester-TMRE (Thermo Fisher Scientific, Waltham, MA, USA) for mitochondrial membrane potential. For the analyses of cell cycle distribution, cells were fixed in 70% ethanol and then stained with PI. All data were acquired using the flow cytometer MACSQuant Analyzer (Miltenyi Biotec, Bergisch Gladbach, Germany) and analyzed with the MACSQuantify software version 2.11 (Miltenyi Biotec, Bergisch Gladbach, Germany).

### 4.5. Western Blotting

Cell lysates, SDS-PAGE and Western blot were performed as described in [5]. Antibodies used were: ATM p-Ser1981 #13050, Chk2 p-Thr68 #2661, Chk2 #2662, Chk1 p-Ser345 #2341, Chk1 #2360, KAP1 #5868, cleaved caspase 3 (Asp175) #9661 (Cell Signaling Technology, Danvers, MA, USA), KAP1 p-Ser473 #644602 (Biolegend, San Diego, CA, USA), ATM #AF1655 (R&D Systems Bio-Techne, Minneapolis, MN, USA), vinculin #V9131, β-actin #A2066 (Millipore Sigma, Burlington, MA, USA). Detection was performed by exposure to standard X-ray films or by a CCD camera system (UVITEC, Cambridge, UK).

### 4.6. Mutational Profiling of Cell Lines

Genomic DNA was extracted using the Nucleospin Tissue kit (Macherey-Nagel GmbH & Co, Düren, Germany) and quantified using Qubit 2.0 and the Qubit DNA HS Assay kit (Thermo Fisher Scientific, Waltham, MA, USA). DNA was run on the Oncomine Comprehensive Assay (OCA) Plus targeted panel of 1.7 Mb (Thermo Fisher Scientific, Waltham, MA, USA) with an AmpliSeq-based enrichment. All library preparation was performed manually according to manufacturer’s instructions (MAN0018490). Multiplex PCR amplification was conducted using 20 ng of DNA as input. Purified libraries were quantified with real-time PCR with the Ion Library TaqMan™ Quantitation Kit (Thermo Fisher Scientific, Waltham, MA, USA). The 50 pM libraries were pooled and loaded onto Ion 550™ Chips (Thermo Fisher Scientific, Waltham, MA, USA) according to manufacturer’s instructions (MAN0017275) and prepared for sequencing using the Ion Chef™ System (Thermo Fisher Scientific, Waltham, MA, USA). Sequencing was performed using the Ion Gene Studio S5 Sequencer (Thermo Fisher Scientific, Waltham, MA, USA).

For the analysis, data were initially processed using Ion Torrent Suite Software™ (Thermo Fisher Scientific, Waltham, MA, USA), and variant calling was performed using the Variant Caller plugin. Variants were filtered for coverage greater than 40 reads, frequency greater than 5%, quality value greater than 30, and coverage depth greater than 500X. Resulting variants were annotated using the OpenCravat tool (available online: https://opencravat.org (accessed on 14 February 2022)) and the Ion Reporter™ Software (v. 5.18, Thermo Fisher Scientific, Waltham, MA, USA). Variants were classified using ClinVar (available online: https://www.ncbi.nlm.nih.gov (accessed on 14 February 2022)), cBioPortal (available online: https://www.cbioportal.org (accessed on 14 February 2022)), and dbSNP databases. Variants categorized as neutral/benign and variants with a frequency >0.00001 in the population (not pathogenic) were considered as single nucleotide polymorphisms (SNPs) and were thus excluded.

### 4.7. Statistical Analyses

Data are expressed as the mean ± standard deviation (SD) of independent experiments. GraphPad Prism 9 software (GraphPad Inc, San Diego, CA, USA) was used to perform graphs and statistical analyses. Specifically, one-way ANOVA test was used to calculate significance of cell viability, cell death, and TMRE assays. Tukey post hoc test was applied. Two-way ANOVA followed by Bonferroni posttest was used to calculate the significance of cell cycle experiments. For γH2AX and 53BP1 foci enumeration, *p* values were calculated with Student’s *t* test.

## Data Availability

The data presented in this study are available in article and Supplementary Materials and original files are available on request from the corresponding author.

## Supplementary Materials

The following supporting information can be downloaded at: https://www.mdpi.com/article/10.3390/ijms23073834/s1.
