# Supplementary figures and images for: Targeting the DNA Damage Response to Increase Anthracycline-Based Chemotherapy Cytotoxicity in T-Cell Lymphoma
Source: Int J Mol Sci. 2022 Mar 30;23(7):3834. doi: 10.3390/ijms23073834 (PMC8999036; doi:10.3390/ijms23073834)

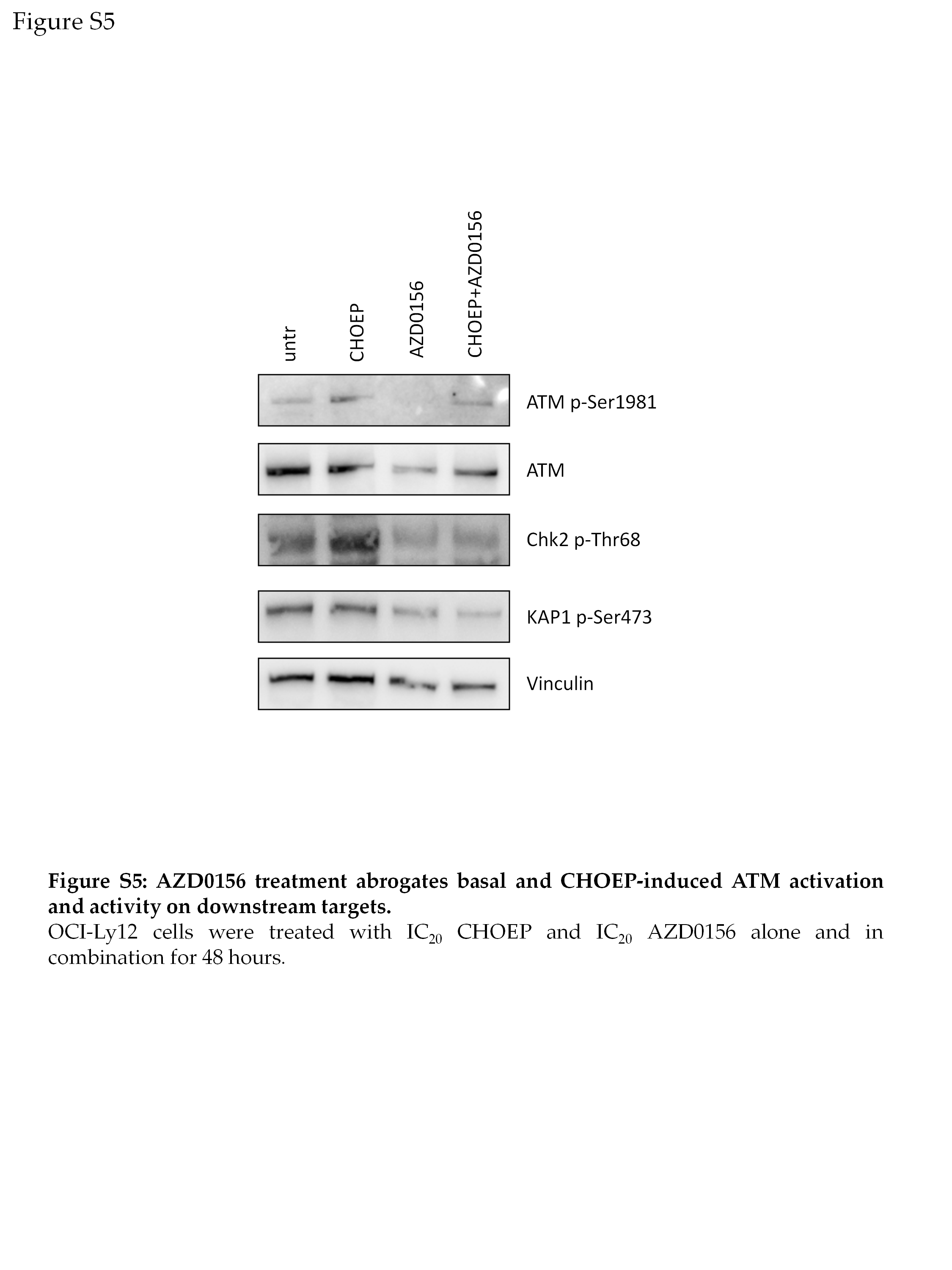

Supplement: Supplementary file 1 [file ijms-23-03834-s001.zip › Figure S5.TIF]

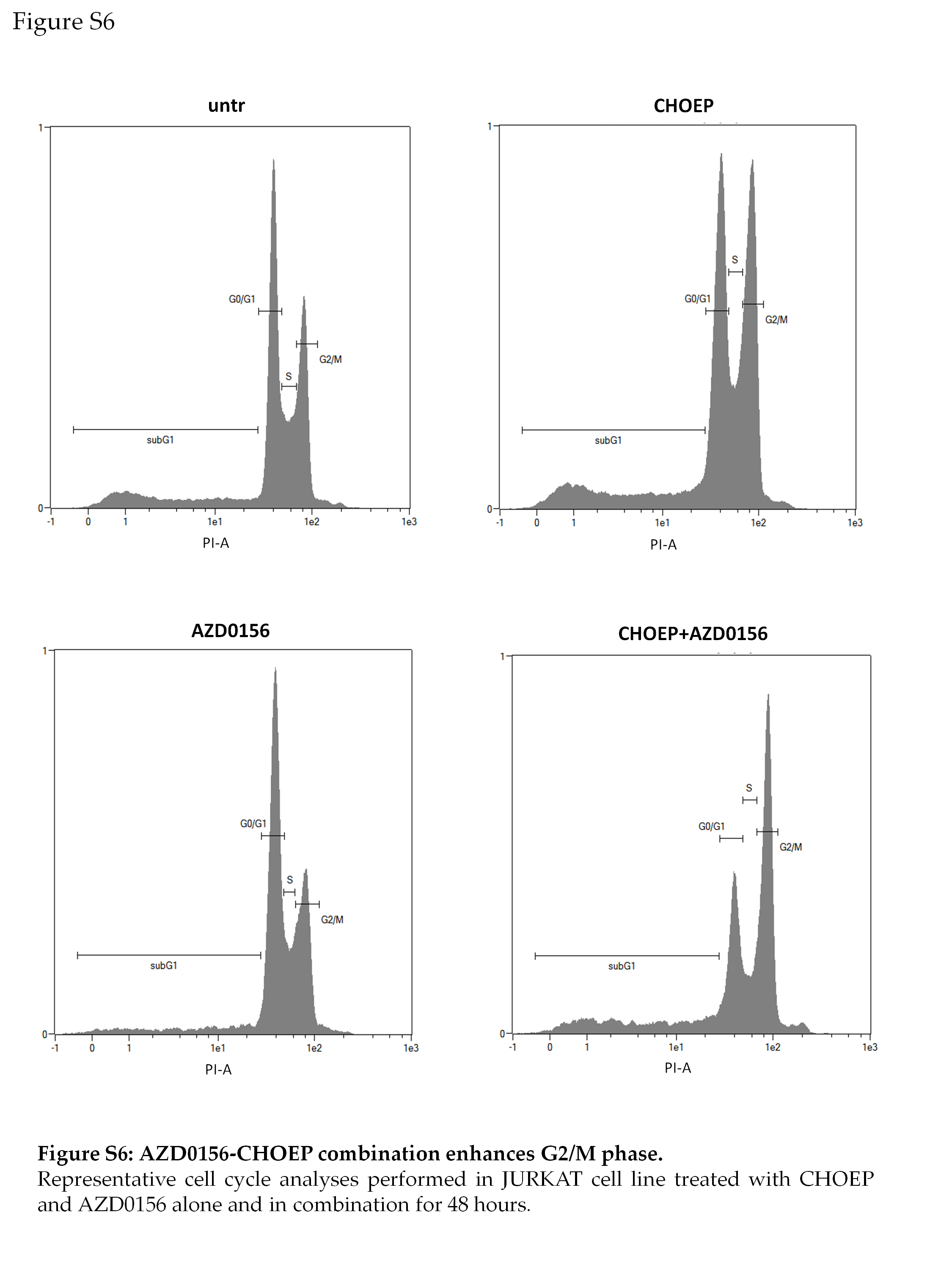

Supplement: Supplementary file 1 [file ijms-23-03834-s001.zip › Figure S6.TIF]

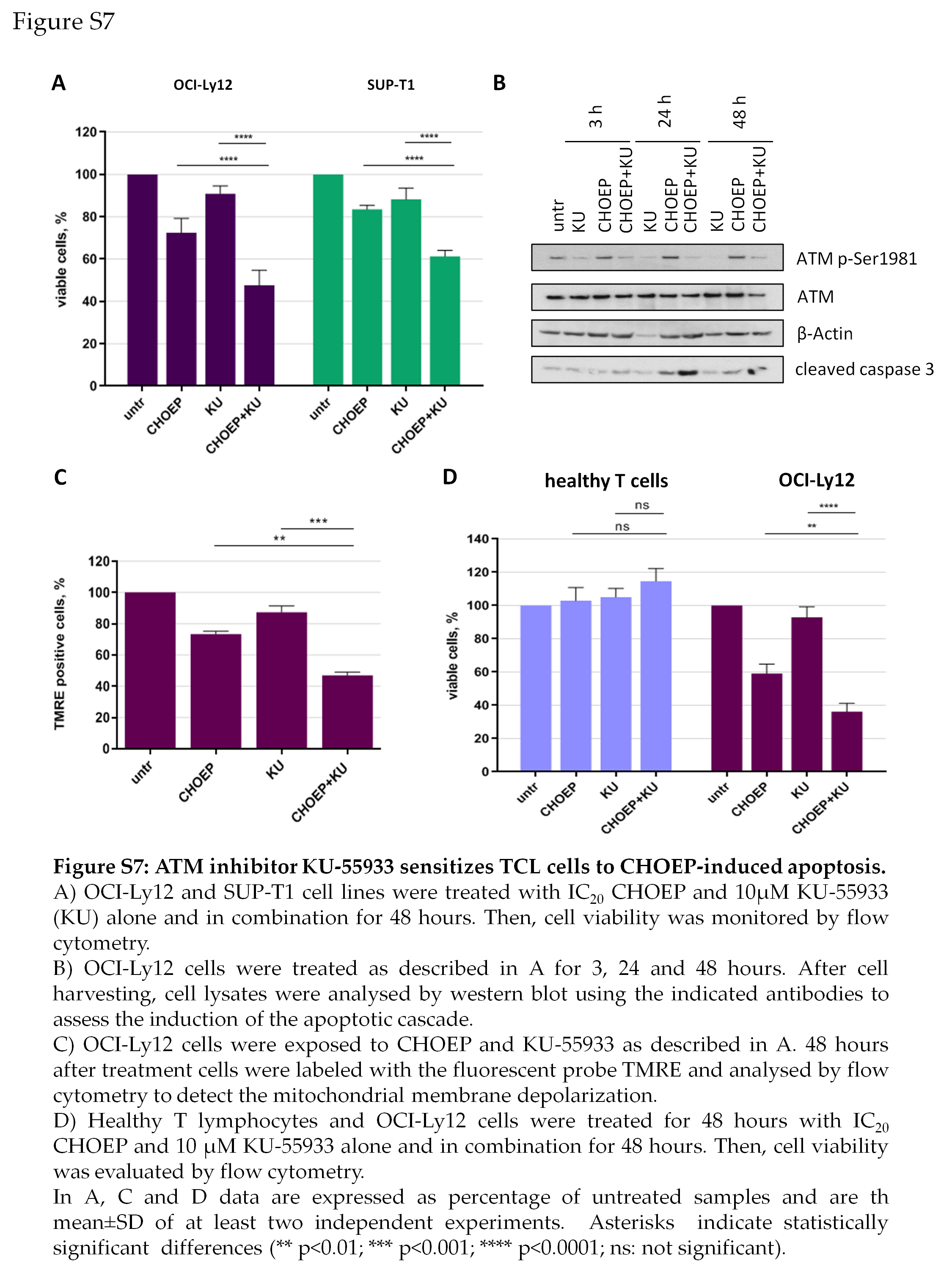

Supplement: Supplementary file 1 [file ijms-23-03834-s001.zip › Figure S7.TIF]

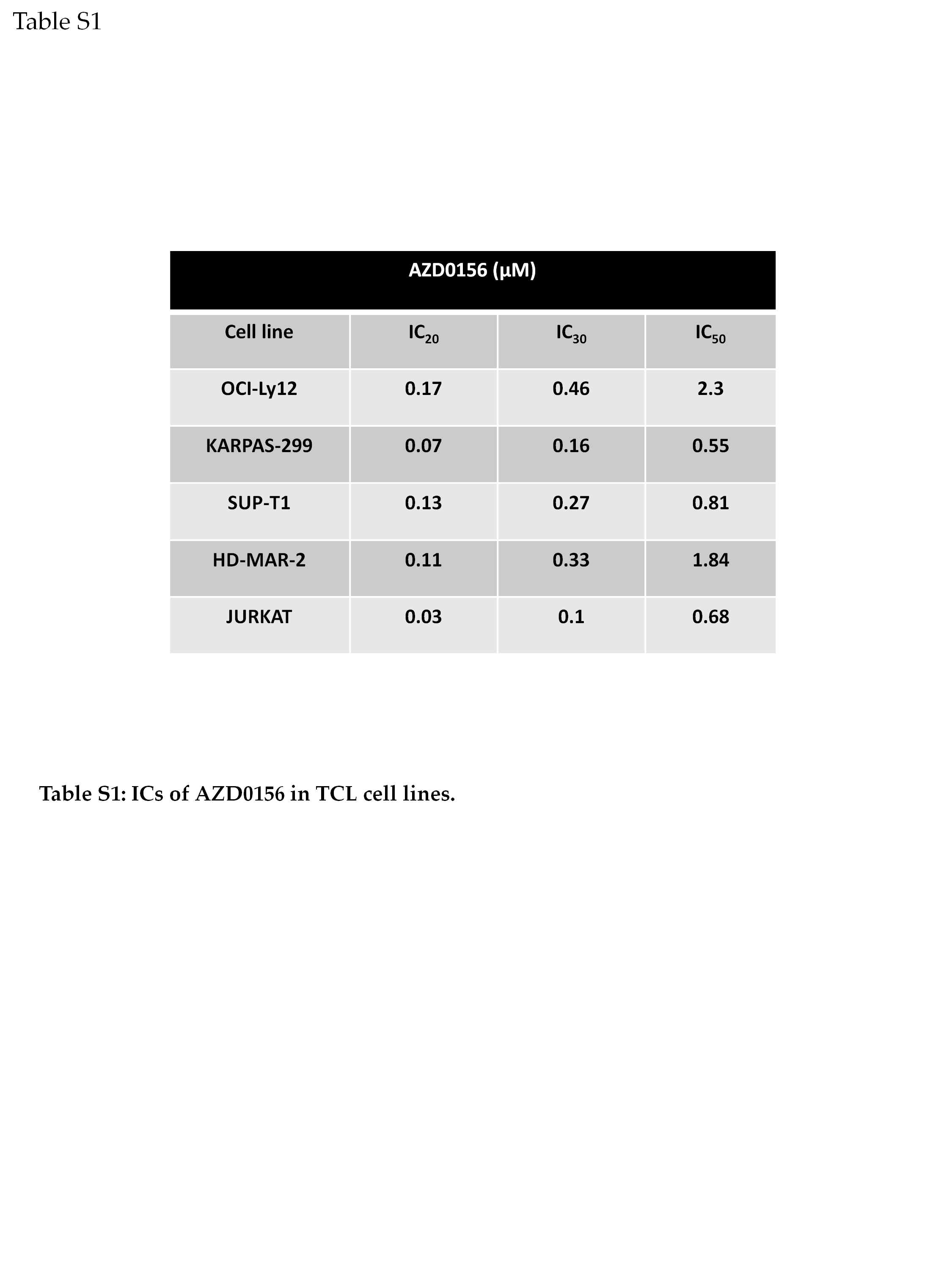

Supplement: Supplementary file 1 [file ijms-23-03834-s001.zip › Table S1.TIF]

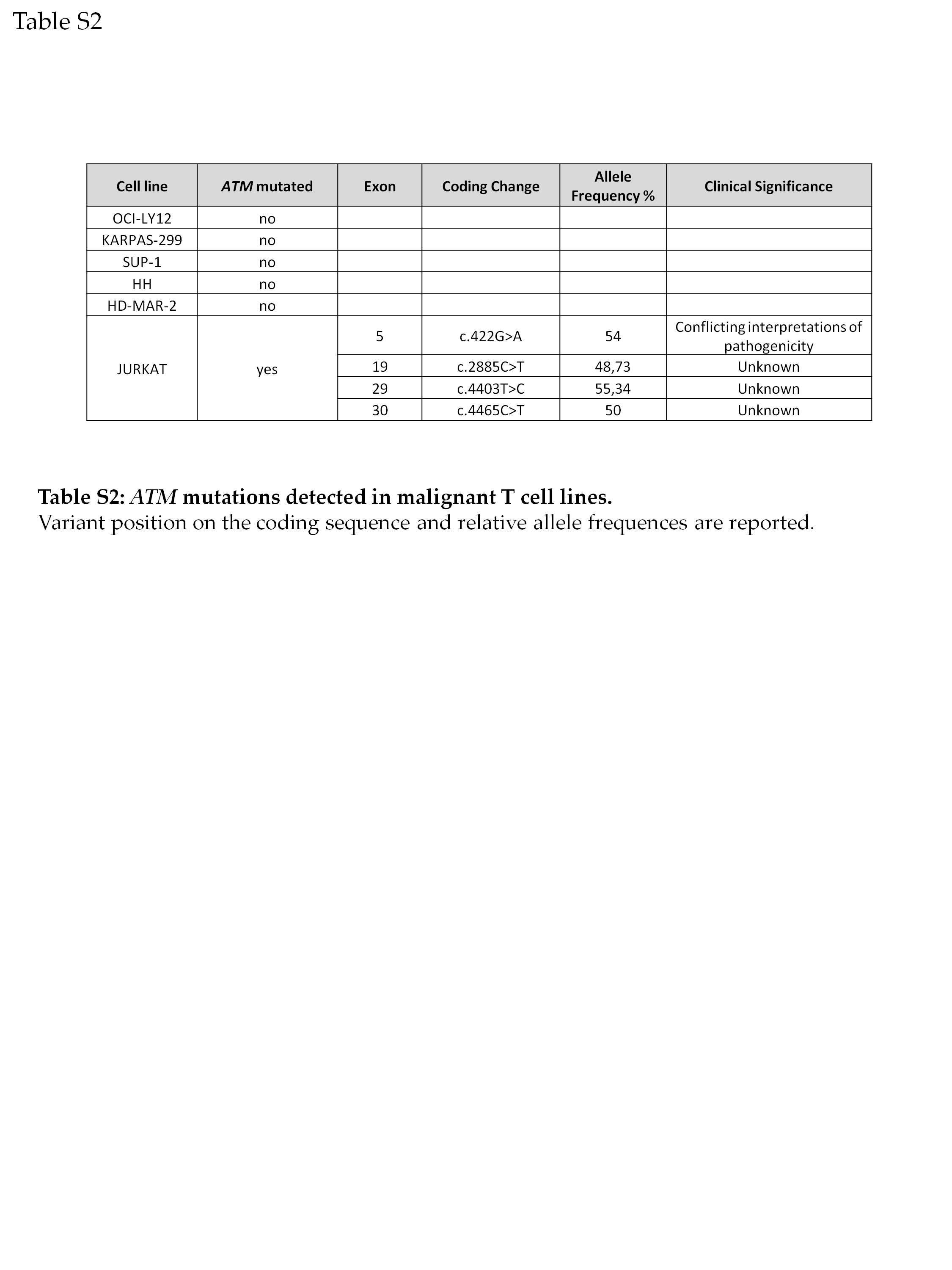

Supplement: Supplementary file 1 [file ijms-23-03834-s001.zip › Table S2.TIF]

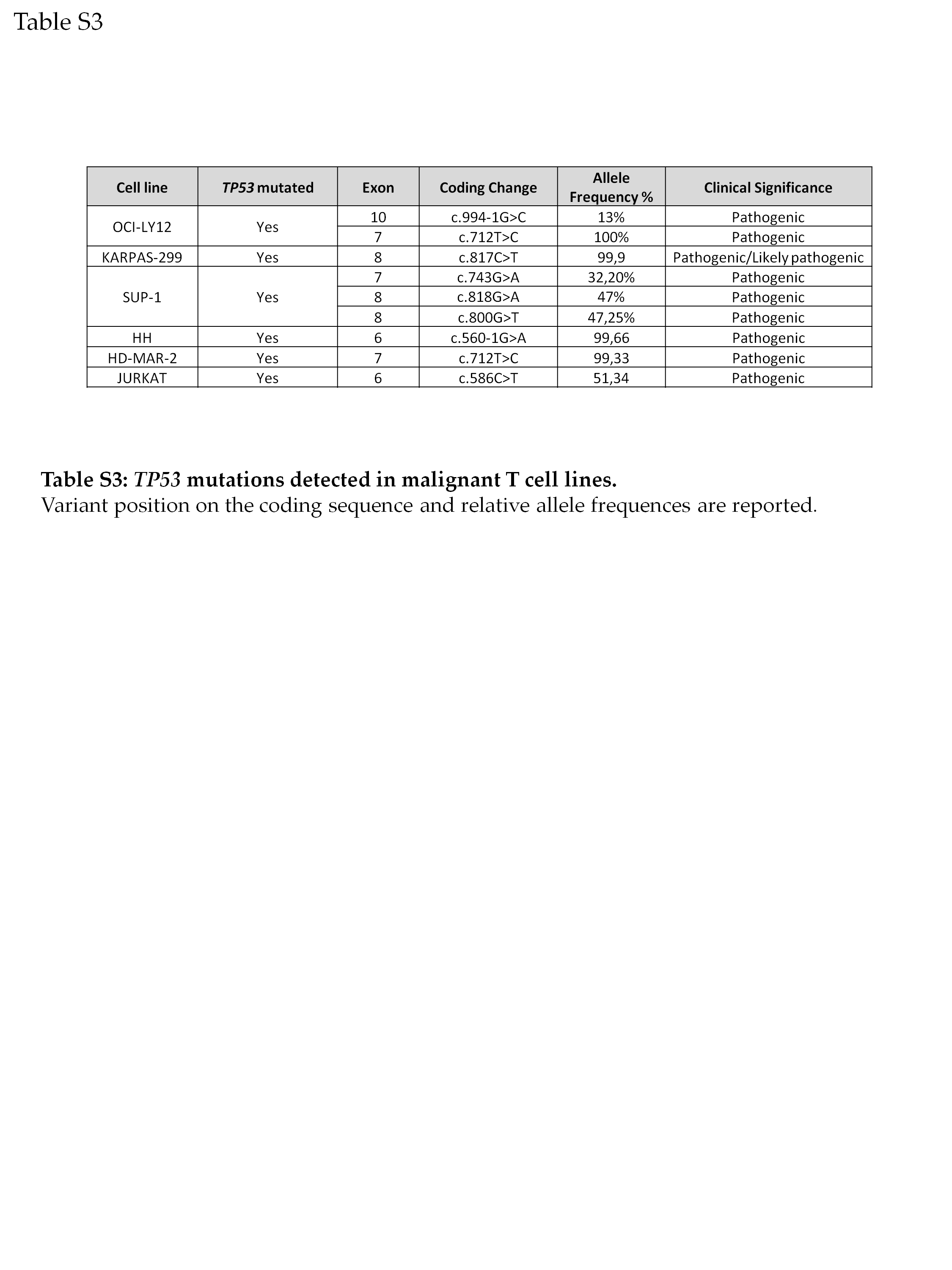

Supplement: Supplementary file 1 [file ijms-23-03834-s001.zip › Table S3.TIF]

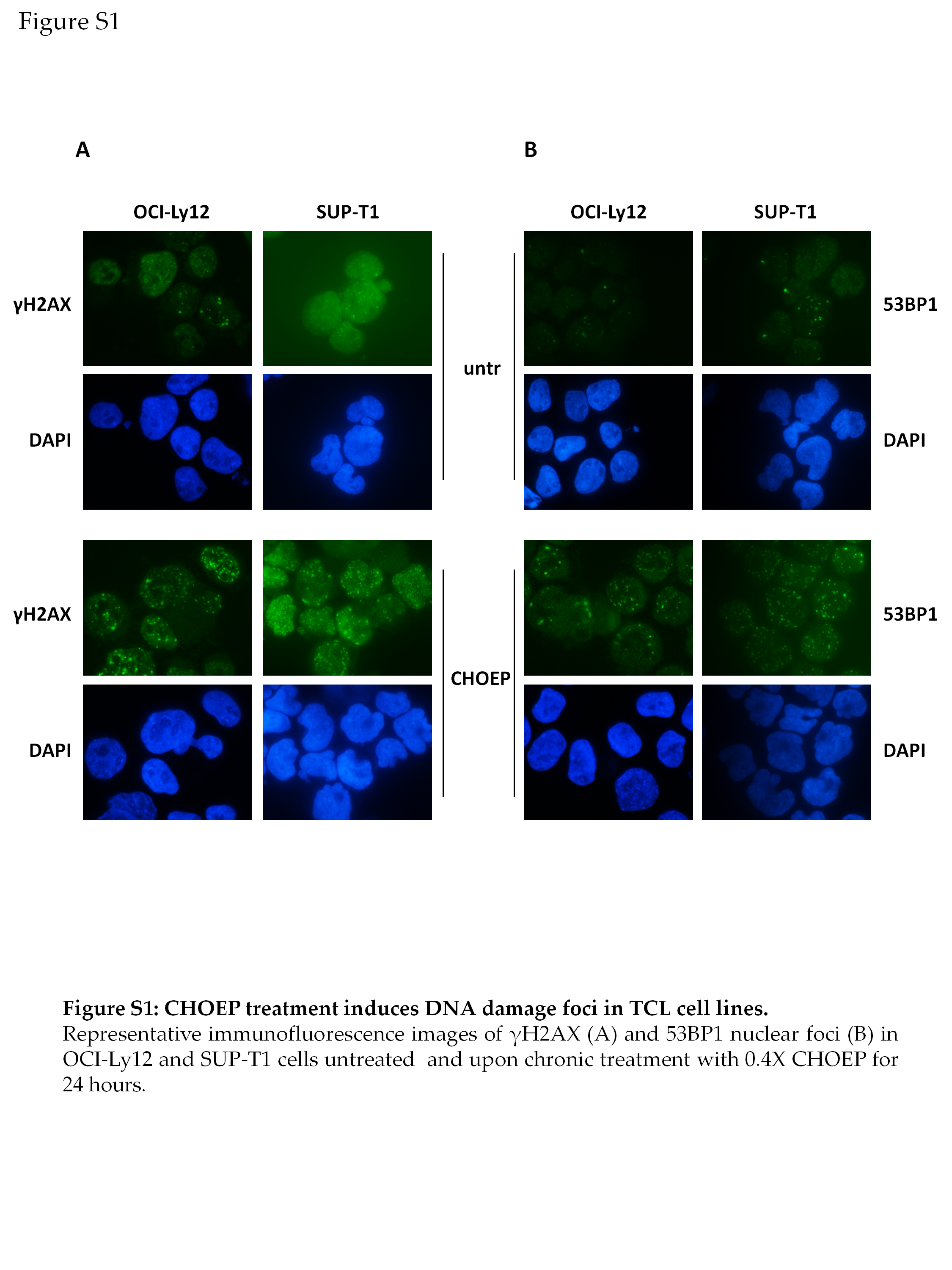

Supplement: Supplementary file 1 [file ijms-23-03834-s001.zip › Figure S1.TIF]

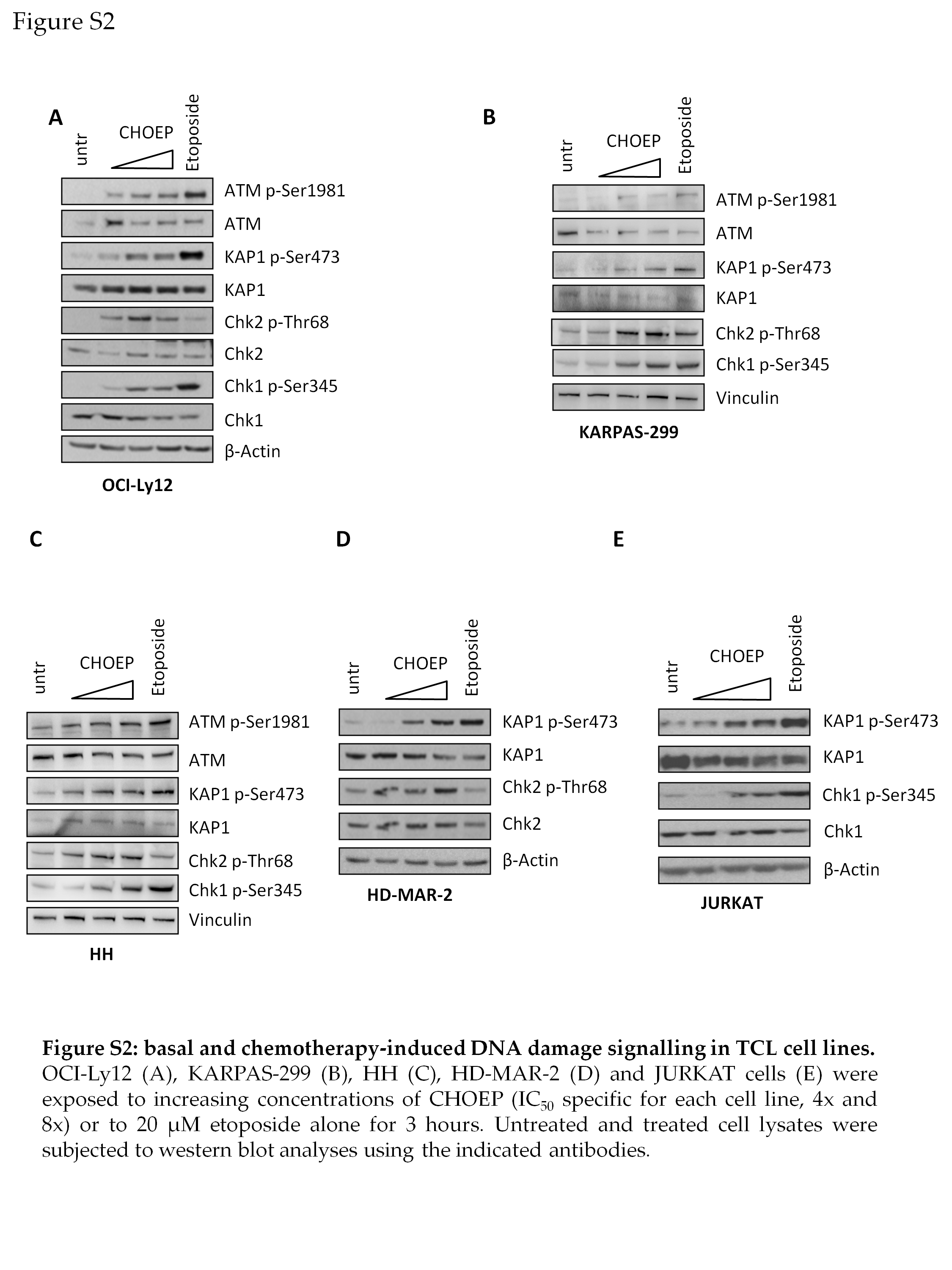

Supplement: Supplementary file 1 [file ijms-23-03834-s001.zip › Figure S2.TIF]

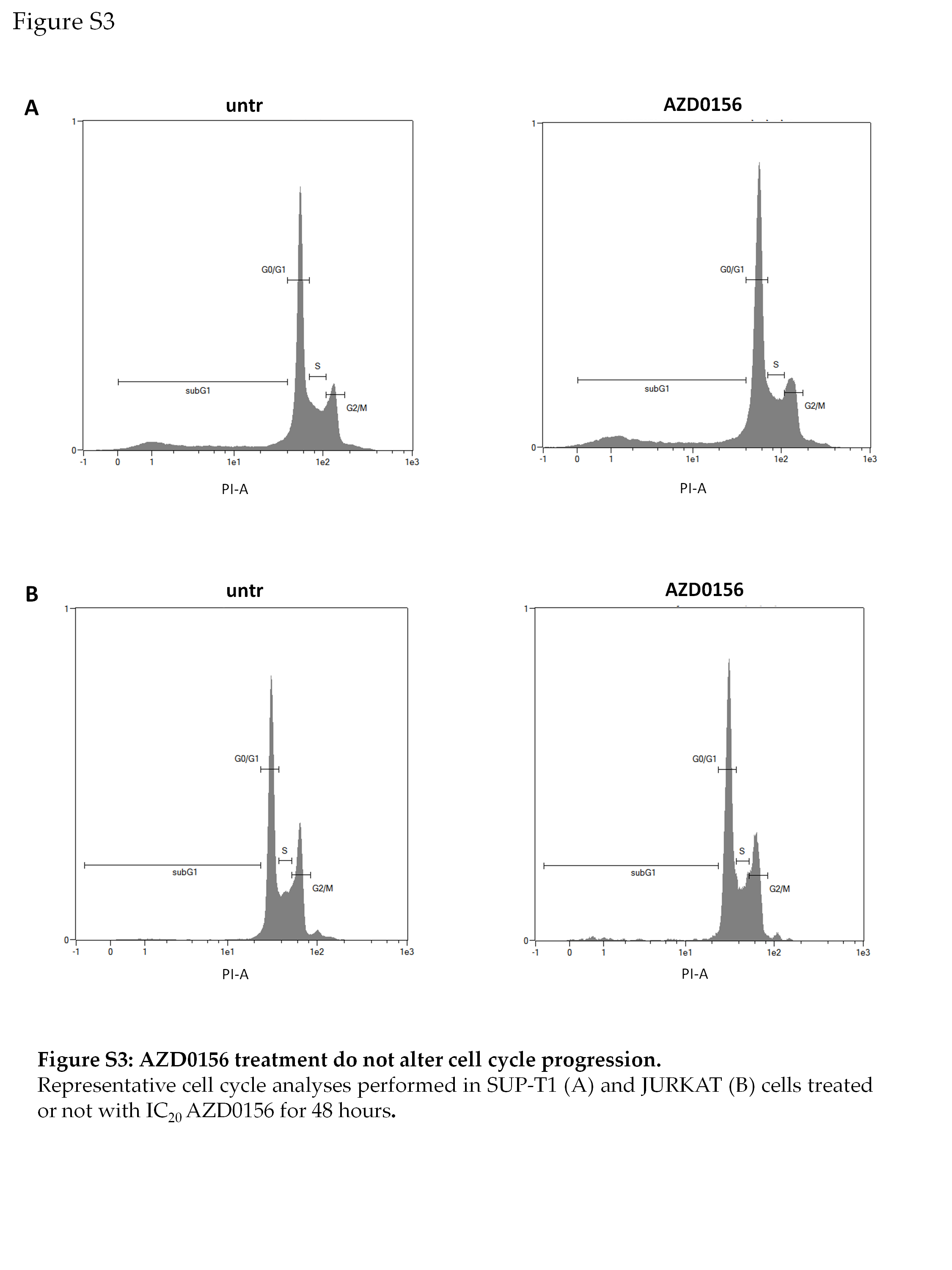

Supplement: Supplementary file 1 [file ijms-23-03834-s001.zip › Figure S3.TIF]

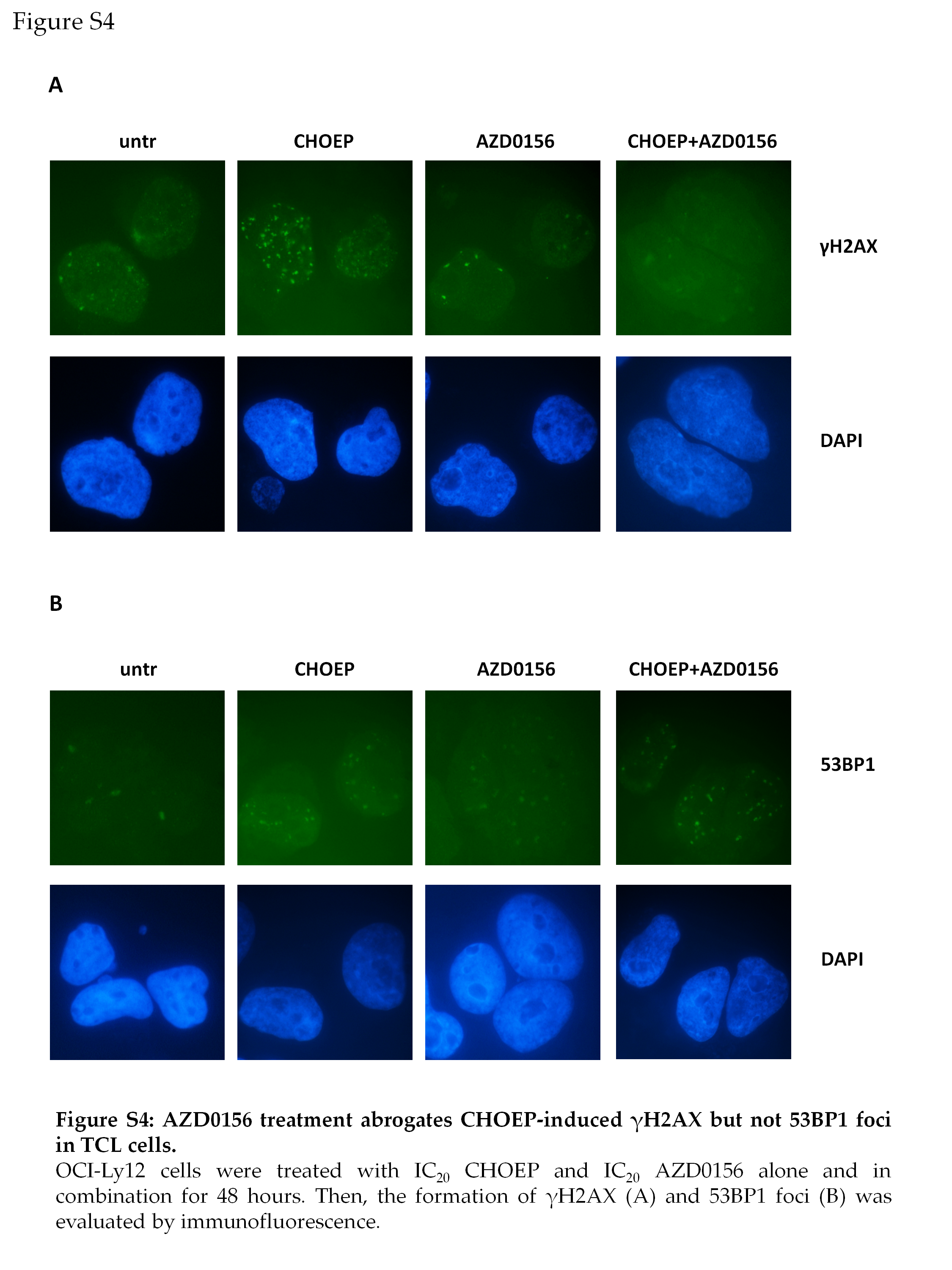

Supplement: Supplementary file 1 [file ijms-23-03834-s001.zip › Figure S4.TIF]
